# Adult brain neurons require continual expression of the schizophrenia-risk gene Tcf4 for structural and functional integrity

**DOI:** 10.1038/s41398-021-01618-x

**Published:** 2021-09-25

**Authors:** Dipannita Sarkar, Mohammad Shariq, Deepanjali Dwivedi, Nirmal Krishnan, Ronald Naumann, Upinder Singh Bhalla, Hiyaa Singhee Ghosh

**Affiliations:** 1 National Center for Biological Sciences, Tata Institute of Fundamental Research, Bangalore, 560065 India; 2 The University of Trans-Disciplinary Health Sciences and Technology, Bangalore, 560064 India; 3 MPI of Molecular Cell Biology and Genetics, Dresden, 01307 Germany

**Keywords:** Molecular neuroscience, Schizophrenia

## Abstract

The schizophrenia-risk gene Tcf4 has been widely studied in the context of brain development using mouse models of haploinsufficiency, in utero knockdown and embryonic deletion. However, Tcf4 continues to be abundantly expressed in adult brain neurons where its functions remain unknown. Given the importance of Tcf4 in psychiatric diseases, we investigated its role in adult neurons using cell-specific deletion and genetic tracing in adult animals. Acute loss of Tcf4 in adult excitatory neurons in vivo caused hyperexcitability and increased dendritic complexity of neurons, effects that were distinct from previously observed effects in embryonic-deficiency models. Interestingly, transcriptomic analysis of genetically traced adult-deleted FACS-sorted Tcf4-knockout neurons revealed that Tcf4 targets in adult neurons are distinct from those in the embryonic brain. Meta-analysis of the adult-deleted neuronal transcriptome from our study with the existing datasets of embryonic Tcf4 deficiencies revealed plasma membrane and ciliary genes to underlie Tcf4-mediated structure-function regulation specifically in adult neurons. The profound changes both in the structure and excitability of adult neurons upon acute loss of Tcf4 indicates that proactive regulation of membrane-related processes underlies the functional and structural integrity of adult neurons. These findings not only provide insights for the functional relevance of continual expression of a psychiatric disease-risk gene in the adult brain but also identify previously unappreciated gene networks underpinning mature neuronal regulation during the adult lifespan.

## Introduction

A large body of literature informs us about the role of transcription factors (TFs) in neural specification and morphogenesis during brain development. However, many TFs continue to be expressed in mature neurons long after neuronal development and throughout the adult lifespan, although our knowledge about the functions of these TF in mature neurons remain sparse. Studies in invertebrates and vertebrates have shown the importance of continual expression of certain TF in fate maintenance of specific neuronal subtypes [1–4]. Beyond the steady-state fate-maintenance role, a substantial body of work has also demonstrated the role of TF-mediated gene regulation in neuronal activity. Focusing on the immediate early response genes (IEG), these studies show that the synaptic changes are communicated to the nucleus to trigger gene regulation that influence downstream processing for memory formation and consolidation [5], thereby establishing the significance of gene regulation in short- and long-term neuronal plasticity. However, beyond the IEG, very little is known about TFs and their role in regulating the structure or function of mature neurons in the adult brain. To this end, we investigated transcription factor 4 (Tcf4) because of its persistent expression in the adult mammalian brain throughout the lifespan and noted association with both neurodevelopmental and psychiatric disorders.

Tcf4 is a basic helix–loop–helix (bHLH) TF implicated in schizophrenia, autism [6–10] and Pitt–Hopkins syndrome [11–14]. Multiple studies in the last several years have highlighted its important role in neuronal development and acquisition of normal brain architecture [15–20]. However, Tcf4 continues to be expressed in rodent and human brain throughout the adult lifespan [19, 21], where its function(s) remain unknown. Since Tcf4 knockout animals are perinatally lethal [22, 23], investigations regarding the effect of Tcf4 loss in the adult brain has been limited to studies in Tcf4-heterozygous animal model, which harbour only one functional allele of Tcf4 throughout the embryonic and postnatal development in all cells [9, 24]. Notably, recent studies demonstrate that the expression of Tcf4 in the adult brain is broad, spanning neurons, astrocytes and oligodendrocytes [25, 26]. This further highlights the complexity in data interpretation from embryonic heterozygous or knockout models, wherein the Tcf4 alteration would be present in all types of cells starting from when the brain is developing, thereby making it difficult to tease apart the primary and secondary effects of Tcf4 deletion in any given cell type and developmental age.

A recent study revealed that Tcf4’s transcriptional activity is in fact regulated by neuronal activity [27], suggesting a potential ongoing function for Tcf4-mediated transcriptional regulation in brain functions since Tcf4 is continually expressed in the adult neurons. However, given Tcf4’s wide expression in many cell types of the adult brain, elucidating its function specifically in adult neurons require an in vivo system which is not only inducible (delete Tcf4 only in adult stage, after the full development of the brain is completed) but also one that is cell-type specific (conditional), so that Tcf4 could be deleted only in a specific cell type, for example, only in neurons. There has been only one study which used a sparse-expressing pan-neuronal Cre line (Slick-V) to delete Tcf4 in sparse neuronal populations showing a reduction of dendritic spines [28]. However, this study was limited to only spine counts, without providing any molecular, electrophysiological or behavioural insights into Tcf4’s role or specific targets in adult neurons. To gain deeper functional insights for continual Tcf4 expression in adult neurons, we used the LoxP-CreERt2 based inducible and conditional deletion system, to delete Tcf4 specifically in the excitatory neurons in the adult brain using the CaMK2αCreERt2 Cre driver, and genetically traced the deleted neurons with fluorescent reporter for molecular analyses in vivo. Through detailed analyses at the molecular, morphological, electrophysiological and behavioural level, we demonstrate that Tcf4 is critically required for normal dendritic structure and excitability of mature neurons. Finally, through transcriptomic analyses of FACS-sorted genetically labelled adult-deleted Tcf4-KO neurons, we identify the unique targets of Tcf4 in adult excitatory neurons, demonstrating that these are vastly distinct from its targets in the embryonic brain. The novel targets of Tcf4 in adult neurons reveal previously unappreciated pathways, such as plasma membrane and cilium-related processes, to underlie the proper structure and function of mature neurons during the adult lifespan.

## Results

### Tcf4 is expressed in excitatory and inhibitory neurons of the adult brain

**Fig. 1:**
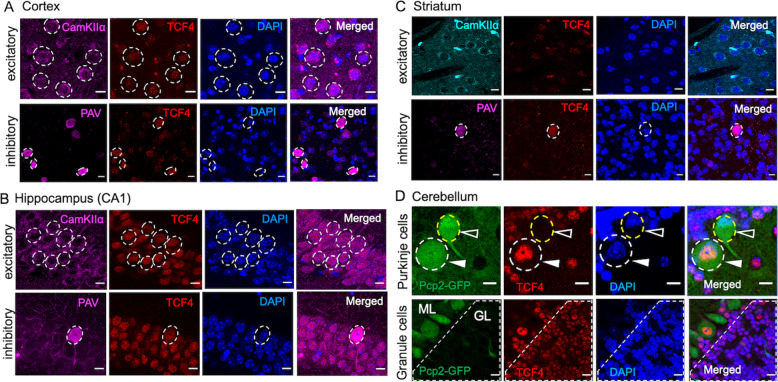
Tcf4 protein expression in excitatory and inhibitory neurons in adult brain. Representative immunofluorescence images showing Tcf4 protein expression in the nucleus of excitatory and inhibitory neurons in **A** cortex, **B** hippocampus (CA1 region), **C** striatum (caudate putamen) and **D** cerebellum; solid arrow: TCF4+ PC, empty arrow: TCF4− PC; PC Purkinje cells, ML molecular layer, GL granule cell layer (shown in dash-lined box), representative of 3–5 adult mice. Scale bar = 10 µm.

Given the wide expression of Tcf4 across the brain parenchyma, in multiple cell types and across developmental and adult lifespan, we focused on neuronal populations for an in-depth characterisation of Tcf4-protein expression in the adult brain parenchyma. A recent study used dual in situ hybridisation to show mRNA transcript co-localisation of Tcf4 with vGlut and vGAT expressing neurons in the prefrontal cortex (PFC), however Tcf4 protein expression was not confirmed in excitatory neurons [26]. We used inhibitory and excitatory neuronal markers in different regions of the adult forebrain and cerebellum to examine Tcf4-protein expression through immunofluorescence-based co-localisation studies. As shown in Figs. 1A–D and S1A, B, we observed prominent Tcf4 expression in both excitatory and inhibitory neurons of the adult brain cortex, hippocampus, striatum and cerebellum. Interestingly, we observed that the parvalbumin positive inhibitory neurons of the dentate gyrus (DG) show higher Tcf4 expression when compared to the excitatory granule cells (GC) in the DG (Fig. S1A). However, in the cortex and CA1, Tcf4 protein expression was comparable between the excitatory and inhibitory neuronal populations (Figs. 1A, B and S1B). While we found high Tcf4 expression in both excitatory and inhibitory neurons in the cortex and hippocampus, in the striatum (caudate putamen), Tcf4 expression was predominantly seen in the inhibitory neurons (Fig. 1C).

We also characterised Tcf4 protein expression in neurons of the cerebellum. For this, we used genetic labelling of Purkinje cells (PC) through a Pcp2-Cre-GFP reporter that is expressed in postnatal PC, and used cell location and morphology to identify GC in the cerebellum. Tcf4 immunostaining showed prominent expression of Tcf4 in the PC as well as in GC (Fig. 1D). Interestingly, we observed that while majority of Purkinje neurons (Figs. 1D and S1C, filled arrows) expressed very high levels of Tcf4 protein, some did not express any Tcf4 (Figs. 1D and S1C, open arrows). This revealed the potential existence of two types of PC in the adult brain cerebellum that could be distinguished as TCF4-positive and TCF4-negative cells. Unlike, the Purkinje neurons, all GC in the adult brain cerebellum expressed Tcf4 protein (Fig. 1D, dashed-line box).

### Tcf4 deletion in adult brain excitatory neurons results in increased baseline cFos activity

Tcf4’s function has been well investigated during brain development, and in conditions of haploinsufficiency (Tcf4-heterozygous mice). To gain insight into the functional relevance for its continual expression in the mature neurons in the adult brain, we used LoxP-Cre based inducible and conditional gene deletion in the excitatory neurons of adult animals, using the CaMK2α-CreERt2 transgenic line [29]. In addition, to track the Tcf4-deleted mature neurons, we employed genetic reporting in our inducible-conditional deletion system by introducing a flox-STOP–flox-EGFP cassette driven by Rosa26 locus, which enabled indelible marking of cells in which Cre was induced upon tamoxifen treatment. This allowed us to detect the Cre-recombined cells in the WT and KO brains because of EGFP expression due to CreERt2 induction.

**Fig. 2:**
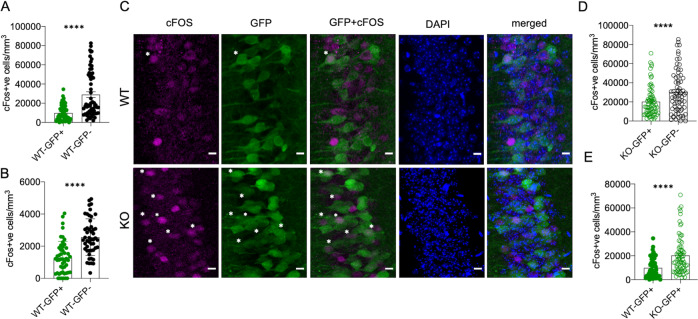
Tcf4 deletion in adult excitatory neurons increases cFos activity. **A**, **B** Quantification of cFos+ cells in GFP+ (SEM+/− 1013) versus GFP− (SEM+/− 3001) CA1 neurons (**A**) and granule neurons in DG (**B**), (GFP+ : SEM+/− 146.6), (GFP−: SEM+/− 162.7) of wild-type adult animals (~P60–90). **C** Representative images of CA1 neurons showing cFos expression. Asterisks depict cFos+GFP+ cells. **D** Quantification of cFos+ cells in GFP+ versus GFP− CA1 neurons of Tcf4-KO brain, (GFP+ : SEM+/− 1887), (GFP−: SEM+/− 2677). **E** Quantification of cFos+ cells in GFP+ CA1 neurons from WT and KO brains, (WT: SEM+/− 1013), (KO: SEM+/− 1887) (each dot represents a brain section, ~10 sections per brain were quantified, CA1: 7 WT, 8 KO and DG: 5 WT, 5 KO animals), Wilcoxon test, ****p < 0.0001, error bar represents SEM. Analysis was done 3 weeks post deletion. Scale bar = 10 µm.

After confirming CaMK2αCreERt2 induction in mature neurons detected by GFP expression, we first analysed baseline cFos expression, a widely used molecular indicator of neuronal activity [30], for the GFP-positive (GFP+) and GFP-negative (GFP−) cells in WT (tamoxifen-treated CaMK2αCreERt2; stop-flox-EGFP; Tcf4^+/+^) animals. Interestingly, we observed that the baseline cFos expression of GFP+ (i.e., CaMK2α+) and GFP− (i.e., CaMK2α−) CA1 neurons in the WT brain was different: the GFP− CA1 neurons were more frequently cFos positive when compared to the GFP+ CA1 neurons (Figs. 2A, C, upper panel, S2A, H). This suggests that CA1 neurons can be distinguished as two populations with different baseline activity. The GFP+/CaMK2α+ based distinction of baseline activity also held true for granule neurons of the DG in WT brains, wherein the GFP− cells were more frequently cFos+ than the GFP+ GC (Figs. 2B and S2B).

Next, we examined the functional relevance of Tcf4 expression in CaMK2α+ mature excitatory neurons by inducing Tcf4 deletion in these neurons in the adult mice. For this, we used a Tcf4-flox mouse line used previously [31, 32]. In this Tcf4-flox line [31], the bHLH domain of the Tcf4 locus is flanked by the LoxP sites, as depicted in the schematic (Fig. S2C). This leads to functional deletion of Tcf4 upon Cre-mediated recombination, since TCF4 requires the bHLH domain to bind to DNA and regulate transcription. In this deletion model, a truncated protein is formed from the N-terminal part of the Tcf4 locus preceding the bHLH region, the presence of which can be detected as a mark of functional Tcf4 deletion. We first confirmed deletion of Tcf4 in our mice through recombination PCR after Cre induction in adult animals (~P60), which showed LoxP recombination for Tcf4 locus only in the KO brains (tamoxifen-treated CaMK2αCreERt2; stop-EGFP; Tcf4^fl/fl^) and not in WT brains (tamoxifen-treated CaMK2αCreERt2; stop-EGFP; Tcf4^+/+^) (Fig. S2D). Further, we also performed a western blot analysis which showed the full length and shorter isoforms of TCF4 protein in WT, whereas only truncated TCF4 in the KO brain (Fig. S2E). For analysing the functional effect of Tcf4 in mature excitatory neurons, we focused our analyses in the CA1 region, because CaMK2α+ is more prevalent in the hippocampus [33], and because of the ease of electrophysiological and morphological studies in the CA1 region. Interestingly, when we compared cFos expression of the GFP+ and GFP− CA1 neurons in the KO brains, we observed that upon acute loss of Tcf4, the GFP+ CA1 neurons became increasingly more cFos positive, thereby reducing the frequency difference between GFP− cFos+ and GFP+ cFos+ cell populations (Figs. 2C lower panel, D and S2F). Consistently, the GFP+ cells in KO brain were more frequently cFos+ when compared with GFP+ cells from the WT brains (Figs. 2E and S2G, H). Thus, through genetic tracing of CA1 neurons in which Tcf4 was deleted during adulthood, we demonstrate that continual expression of Tcf4 in CaMK2α+ mature neurons is required for suppressing hyperactivity and maintaining the baseline activity of these neurons in the steady-state adult brain.

### Tcf4 deletion in adult brain excitatory neurons causes changes in membrane properties resulting in hyperexcitability of CA1 neurons

**Fig. 3:**
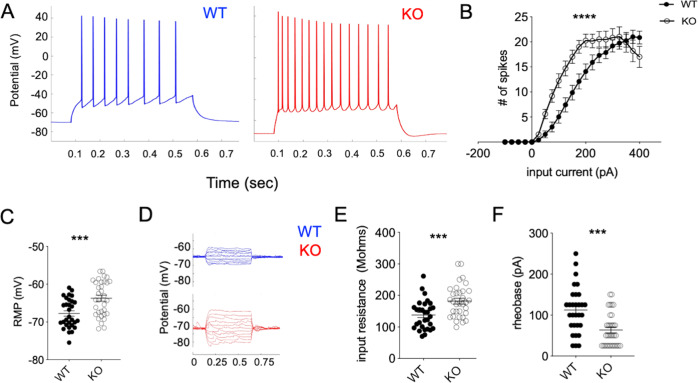
Tcf4 deletion in adult excitatory neuron results in hyperexcitability. **A** Representative traces for action potential spikes from CA1 neurons of WT and KO brains. **B** *F*–*I* curve for firing (two-way ANOVA, ****p < 0.0001, error bar represents SEM). **C**–**F** Cell-intrinsic properties of CA1 neurons. **C** Resting membrane potential (WT: SEM+/− 0.7, KO: SEM+/− 0.8). **D** Input resistance traces (WT: SEM+/− 8.3, KO: SEM+/− 8.9) and **E** quantification. **F** Rheobase (WT: SEM+/− 10.36, KO: SEM+/− 7). Recording from 27 neurons from WT and 33 neurons from KO brains 2–3 weeks post deletion, representing ~5–6 mice per genotype. Mann–Whitney test: ***p < 0.0005, ****p < 0.0001).

To further gain insight into the membrane properties of CA1 neurons after Tcf4 deletion, we performed electrophysiology from brain slices of WT and KO animals. Previous studies targeting medial PFC for Tcf4 knockdown in prenatal brains (E16 in utero knockdown) [19] showed that Tcf4 depletion during corticogenesis results in a reduction of action potential (AP) frequency in juvenile cortical neurons. Since the developing brain neurons have different electrical properties compared to mature neurons in the adult brain, we examined the effect on AP after Tcf4 deletion in mature neurons in the adult brain. Our results show that the loss of Tcf4 in adulthood in fact increases firing in CA1 neurons within ~3 weeks post deletion (Fig. 3A). This was indeed consistent with our cFos data that indicated a higher baseline activity of CA1 neurons upon Tcf4 deletion. We also measured the frequency of AP in response to a series of input currents, and observed that for any given current input, the KO brains fired significantly more AP (Fig. 3B). However, the spike amplitude in the KO brain was lower (Fig. S3A, B).

We next examined multiple cell-intrinsic parameters of the CA1 neurons in the WT and KO brains by whole-cell electrophysiology. Consistent with higher AP, the CA1 neurons in the KO brains were significantly more depolarised when compared to WT, as shown in Fig. 3C with significantly higher resting membrane potential. Also, as shown in Fig. 3D, E, the input resistance (IR) was significantly increased in KO, consistent with higher AP. Furthermore, rheobase, which is the minimum current required for a cell to produce an AP, showed a significant reduction in the KO brains as shown in Fig. 3F. We also examined *I*_*h*_ currents using a hyperpolarizing pulse of 250 pA in current-clamp mode. As shown in Fig. S3C, D, *I*_*h*_ currents as measured by % sag were significantly greater in KO brains. Overall, these observations demonstrate that Tcf4 plays a critical role in maintaining the homoeostatic electrical properties of excitatory neurons in the adult brain, in absence of which these neurons become hyperexcitable.

### Tcf4 deletion in adult brain excitatory neurons results in increased branching and spine counts in CA1 neurons

**Fig. 4:**
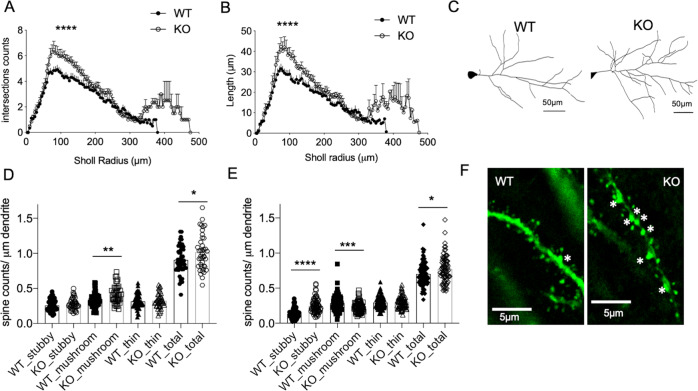
Tcf4 deletion in adult excitatory neurons results in morphological alterations. **A**, **B** Sholl analysis of apical dendrite of CA1 neurons, showing number of intersection (**A**) and length (**B**) per Sholl radii 2 months post deletion. Twenty-six neurons per genotype from 3 WT and 3 KO mice. Two-way ANOVA, ***p < 0.0001, error bar represents SEM. **C** Representative traces of apical dendrites of CA1 neurons 2 months post deletion. **D**, **E** Spine analysis of CA1 apical dendrites at 3 weeks (+/−SEM: 0.01–0.02) (**D**) and 2 months (+/−SEM: 0.008–0.02) (**E**) post deletion, each dot represents counts from a dendritic segment from 3 WT, 4 KO mice for 3 weeks and 3 WT, 3 KO mice for 2 months. **F** Representative image showing spine in CA1 neurons 2 months post deletion, white asterisks mark stubby spines. (Student’s *t* test, ****p < 0.0001, ***p = 0.0006, **p = 0.002, *p = 0.017).

We next examined if Tcf4 deletion in mature CA1 neuron influenced its morphology. For this, we crossed the CaMK2α-CreERt2; Tcf4 wt or flox mice with the sparse-expressing Thy1-GFP line M [34] that enables visualisation and tracing of individual dendritic branches of neurons in brain slices. We induced Tcf4 deletion in these mice in adulthood through tamoxifen administration and analysed morphology of the apical dendrites of CA1 neurons in WT (CaMK2αCreERt2; Thy1-GFP; Tcf4^+/+^) and KO (CaMK2αCreERt2; Thy1-GFP; Tcf4^fl/fl^) animals at 3 weeks post deletion, a timepoint at which the cFos analysis and electrophysiological studies were done. The morphometry data revealed that Tcf4 deletion leads to an alteration of dendritic structure of CA1 neurons as early as 3 weeks post deletion, as indicated by higher number of intersections and length per Sholl radii in KO brains (Fig. S4A–C). We repeated this analysis at 2 months post deletion, which further showed that the increased dendritic complexity due to acute loss of Tcf4 in CA1 neurons remained stable at least up to this timepoint (Fig. 4A–C). Interestingly, a previous report in neonatal brains observed reduction of dendritic processes in the neocortex of Tcf4-heterozygous developing brains at P15 [18], indicating potentially different implications of Tcf4 deficiency based on developmental age and gene dosage. However, consistent with our observations, another study which used lentiviral transduced knockdown of Tcf4 in adult mouse olfactory bulb, observed increased dendritic arborisation in mature interneurons of adult brain after Tcf4 knockdown [35]. These results together reveal that the continual expression of Tcf4 in neurons of the adult brain is not only important for maintaining baseline activity of CA1 neurons but is also important for the structural integrity of mature neurons.

Given the hyperexcitability and increased branching of CA1 neurons as a result of acute Tcf4 loss, we further probed spine densities in these neurons since spines serve as an important functional unit of neural activity. We quantified the density and types of spines in the CA1 neurons at an early (3 weeks post deletion) and late (2 months post deletion) timepoint post deletion. These results revealed an initial modest increase in the density of mushroom spines in the KO brains at ~3 weeks post deletion (Figs. 4D and S4D, E); however, by 2 months post deletion, the alterations in spines became more prominent, with an increase in stubby spines, a modest decrease in the mushroom spines, and an overall increase in total spine density in KO brains (Figs. 4E, F and S4F). Stubby spines are found predominantly during the postnatal development period, a time when neuronal excitability is known to be higher in comparison to mature neurons [36]. Thus, the increase in the density of stubby spines observed in the KO brains is consistent with the hyperexcitability observed in CA1 neurons after Tcf4 deletion and may underlie Tcf4’s regulation of neuronal activity.

Interestingly, a recent study which used a sparse-expressing pan-neuronal Cre line (Slick-V) to delete Tcf4 in all types of adult neurons showed a reduction of dendritic spines [28]. Although different from our observations, possibly due to ‘excitatory’ versus ‘pan’ neuronal nature of Tcf4 deletion which could lead to compensatory effects in the later, these observations are consistent with a role for Tcf4 in synaptic plasticity in adult neurons.

### Tcf4 deletion in adult brain excitatory neurons results in memory deficits

Given the dramatic changes in electrical and morphological properties of CA1 neurons after acute loss of Tcf4, we tested its effects on cognition and memory. Since Tcf4 is implicated in autism spectrum disorder, we first tested social behaviour wherein we assessed (a) sociable nature and (b) short-term social memory. For sociability test, the mice were put in a Y-maze (Fig. S5A), in which one of the two short arms had a dummy mouse to represent an inanimate object, while the other arm had a live mouse, previously unknown to the test animal. The interaction time with the inanimate dummy mouse versus with the live unknown mouse was taken as a proxy for scoring sociability for the animal. As shown in Fig. S5B, the KO animals did not show any deficit in social interaction, clearly preferring the live mouse over the dummy for social interaction, just like the WT animals. Next, we used the same Y-maze to test short-term social memory. For this, the dummy mouse was replaced with a new unfamiliar mouse and the test mouse was allowed to interact with the two mice housed in the two short arms of the Y-maze. Based on whether the test mouse was able to make memory for the mouse it interacted with in the previous session, it should be able to recognise the other mouse in one of the arms on the test-session as ‘new’. As mice have an inherent tendency to prefer novelty for exploration, the interaction time of the test mouse with the ‘novel unfamiliar mouse’ versus ‘previously acquainted-mouse’ could be taken as a proxy for social memory. As shown in Fig. S5C, the Tcf4-KO mice did not show any preference of interaction with the new or the old mouse, indicating that it could not distinguish between the two. The total exploration time during the test did not vary between WT or KO, showing no locomotive or exploration deficit (Fig. S5D). This assay demonstrated that Tcf4 deletion in mature excitatory neuron in the adult brain does not cause sociability deficits but impairs social memory.

We next tested a contextual-fear-memory paradigm to assess fear-memory recall. In the contextual-fear-conditioning (CFC) paradigm, an animal’s ability to remember a fearful context is assessed through its freezing response when brought back into the fearful context. WT and KO animals were first allowed to explore a fear-conditioning chamber and given a mild foot shock at the end of the exploration time. Next day, the animals were brought back into the same chamber, but were not given any foot shock. We observed that while the WT animals showed significant freezing response demonstrating memory recall of its fearful experience in the chamber the prior day, the KO animals showed significantly lower freezing, indicating a deficit in contextual-fear memory (Fig. S5E, F).

We also performed open-field test (OFT) to ascertain if Tcf4 deletion in adult excitatory neurons could have an effect on general activity, given the hyperactive phenotype reported for the Tcf4-heterozygous animals in earlier studies [24, 37]. For this, the mice were allowed to freely explore in an arena, and their movement was recorded for 10 min. Various parameters such as total distance travelled (Fig. S5G), average speed (Fig. S5H) and maximum speed (Fig. S5I) were examined, which showed no difference between the WT and KO animals, indicating no motor deficits.

### Tcf4 suppresses membrane-related gene networks in excitatory mature neurons

**Fig. 5:**
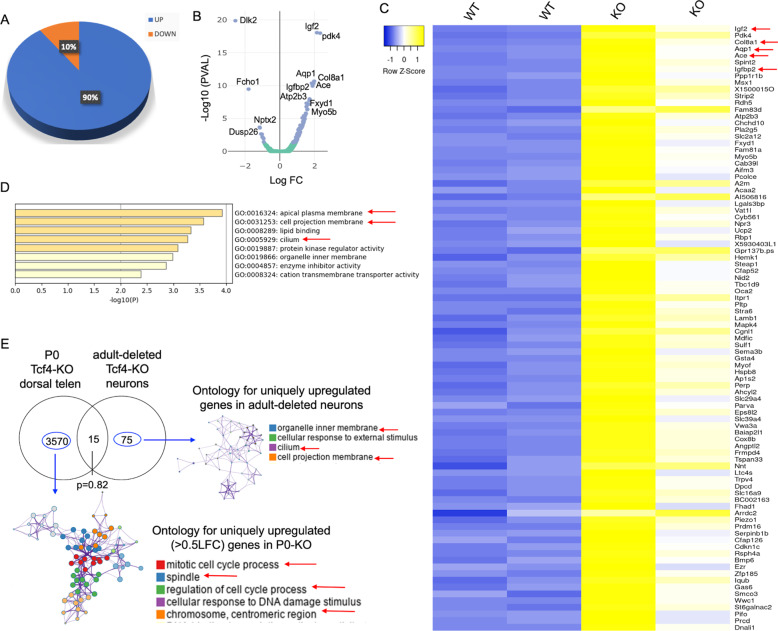
Tcf4 targets membrane-related gene networks in adult neurons. **A** Pie chart showing percentage up- or downregulated genes among total DEG in hippocampal mature neurons after Tcf4 deletion. **B** Volcano plot showing differential gene expression in WT and KO FACS-sorted GFP+ neurons (blue dots represent *p* < 0.05). **C** Heatmap showing upregulated genes in Tcf4-KO neurons, red arrows highlighting growth factor, membrane transport, and ECM-related genes. **D** Gene Ontology analysis of upregulated genes in Tcf4-KO mature neurons, red arrows highlighting plasma membrane and cilia-related GO terms. **E** Meta-analysis of transcriptome from FACS-sorted adult-deleted Tcf4-KO neuron and Tcf4-KO P0 telencephalon showing distinct Tcf4 targets in mature neurons versus developing brain, LFC: log fold change, telen: telencephalon, Fisher’s exact test *p* value = 0.82 shows insignificant overlap.

To understand the molecular underpinnings of the profound structural and functional changes in mature neurons as a result of Tcf4 deletion, we next sought to find the specific targets of Tcf4 in excitatory neurons of the adult brain. For this, we FACS-sorted GFP+ neurons from the hippocampus of WT and KO adult animals 3 weeks post Tcf4 deletion (Fig. S6A), and performed RNA-Seq analysis to identify the differentially expressed genes (DEG) in response to Tcf4 loss in adulthood in mature neurons. The transcriptome analysis identified 100 DEG between the WT and Tcf4-KO neurons, out of which ~90% DEG were upregulated, whereas only ~10 % DEG were downregulated (Figs. 5A and S6B, Supplementary Table 1), indicating an overall suppressive role for Tcf4 on hippocampal excitatory neurons’ gene expression in the adult brain. The top upregulated genes consisted of growth factors, membrane transport, and extracellular matrix (ECM) genes (Fig. 5B, C). Two of the top upregulated genes were insulin-like growth factor 2 (IGF2) and its binding protein IGFBP2. Interestingly, while IGF2 is implicated in spine maturation and stabilisation of presynaptic terminals [38, 39], IGFBP2 has been shown to facilitate intrinsic excitability and neurite branching during early postanal development [40]. To gain insight into the functional impact of Tcf4-mediated gene regulation in excitatory mature neuron, we performed Gene Ontology analysis for the upregulated genes upon Tcf4 deletion. The most prominent functional outcome of the upregulated DEG in Tcf4-deleted mature neurons implicated plasma membrane and cilium-related processes (Fig. 5D). This is consistent with the cellular phenotype observed in our morphological and electrophysiological investigations, which showed dendritic structure and membrane property changes of adult-deleted Tcf4-KO neurons.

### Tcf4 targets in mature neuron are distinct from its targets in the developing brain

Given that Tcf4 is a TF expressed both during development and in the adult brain, we next sought to examine if the Tcf4 targets in ‘developing’ versus ‘mature’ neurons were similar or distinct. A recent study probing Tcf4’s role in cortical brain development performed RNA-Seq analysis of Tcf4-KO dorsal telencephalon cells at P0 [18]. We used this dataset to compare Tcf4 targets in ‘developing’ versus ‘mature’ neurons. For this, first we compared the upregulated genes from adult-deleted Tcf4-KO mature excitatory neurons and P0 Tcf4-KO telencephalon (Fig. 5E). This revealed that ~83% upregulated genes in adult-deleted mature excitatory neurons were unique, and not represented in the ~3585 upregulated genes from P0 Tcf4-KO telencephalon’s (Fig. S6C). Ontology analysis of these uniquely upregulated genes in adult-deleted mature excitatory neurons showed membrane and cilium-related processes as the most enriched ontology cluster (Fig. 5E), confirming that suppression of this gene network is the predominant function of Tcf4-mediated gene regulation in mature neurons in the steady state. In contrast, the unique upregulated genes in the P0-KO telencephalon represented cell-cycle-related genes (Fig. 5E), further demonstrating the distinct influence of Tcf4-mediated gene regulation in embryonic brain and adult neurons.

We next compared our adult-deleted neuronal DEGs with the transcriptome dataset from two studies which analysed micro-dissected CA1 from adult Tcf4-heterozygous animals [9, 24] representing adult hippocampal neurons from animals that have Tcf4 haploinsufficiency throughout development and adulthood. Although, not directly comparable because of Tcf4 heterozygosity and pan-cellular haploinsufficiency in Tcf4-het animals, in contrast to the neuron-specific adult deletion of Tcf4 in our dataset, these Tcf4-Het CA1 transcriptomes were the only transcriptomes available in the adult brain that could reveal possible Tcf4 targets. Comparison of the DEG between adult-deleted Tcf4-KO hippocampal neurons and Tcf4-heterozygous adult CA1 cells from Kennedy et al. showed only 13 commonly upregulated genes (Fig. S6D), whereas the dataset from Phan et al. showed an overlap of only 16 genes (Fig. S6E). Although this overlap was significant (by Fisher’s exact test), this represented only ~14–17% of the upregulated genes observed in adult deletion of Tcf4 in mature neurons. Thus, this meta-analysis data further confirmed that the vast majority of Tcf4 targets in neurons vary depending on whether the deficiency was in adult or embryonic stages, and possibly also on the dosage of the gene (haploinsufficiency versus full deletion). In addition, in the case of Tcf4-heterozygous CA1 cells, there could be secondary effects of pan haploinsufficiency-related defects on the adult Tcf4-haploinsufficient neurons, an effect that can be safely considered to be absent in the case of adult conditional deletion used in our system. Consistent with this, a comparison of Tcf4-het P1 [9] with Tcf4-KO P0 [18] brain also showed minimal overlap of DEG (Fig. S6F), indicating that Tcf4 may regulate different gene networks based on its dosage. To further understand the overall regulatory role of Tcf4, possibly as an activator or suppressor, we also compared the percentage up- or downregulated genes in multiple datasets of adult CA1 and PFC from different Tcf4-mutant mouse models obtained from Phan et al.’s study (Fig. S6G). This analysis did not demonstrate an activator or suppressor role for Tcf4-haploinsufficient brains. Consistent with this, a similar analysis of P0 Tcf4-KO dorsal telencephalon and P1 Tcf4-Het PFC also suggested a bi-directional role of Tcf4 on overall transcription (Fig. S6H). This is in contrast to Tcf4’s role in adult excitatory neurons as shown in our dataset, which suggest an overwhelmingly suppressive role for Tcf4.

Overall, the above meta-analyses of various transcriptomes of Tcf4 deletion and haploinsufficiency revealed that Tcf4 regulates gene expression in an age- and dose-dependent manner. The cell-specific and age-specific deletion analysis from our transcriptome dataset identified a unique set of genes specific to adult excitatory neurons that is kept suppressed by Tcf4, implicating plasma membrane and cilium-related processes as a major regulator of mature neuronal structure and function in the adult brain. This is in contrast with Tcf4’s target genes in developing neocortex, which implicate Tcf4 in activation of neural differentiation while suppressing proliferation [18].

## Discussion

A growing body of work in the past several years has highlighted the important role of gene regulation in neural activity. However, our knowledge about the mediators of gene regulation, the TFs, in the adult neurons remain sparse. To gain more insight into TF function in mature neuron, we focused on Tcf4, whose role in adult brain neurons remain unknown despite its association with multiple neurological diseases. Our results show that Tcf4 expression is continually required in adult neurons to maintain its normal structure and excitability. Acute loss of Tcf4 from mature neurons leads to profound structural and functional changes resulting in higher baseline activity and increased dendritic complexity, leading to cognitive deficits. Transcriptome of Tcf4-deleted mature neurons reveal the unique targets of Tcf4 in adult excitatory neurons, implicating suppression of plasma membrane and cilium-related gene networks to underlie normal structure and function of mature excitatory neurons.

In addition to uncovering an important role for Tcf4 in adult brain neurons, our study also reveals a number of intriguing observations about mature neurons that could set new directions for insights into neuronal cell biology in the adult brain. For instance, our data suggest diversity within a single population of neuron through two examples: firstly, within the CA1 neuronal population, we demonstrate the existence of two subtypes of cells, which could be distinguished on the basis of high and low baseline activity, based on cFos expression. We identified these two populations through indelible marking of CamK2α expression using a genetic reporter. Therefore, the low and high baseline activity of these neurons, as reported by cFos expression, is unlikely to be a dynamic property, and more likely to be specific to CA1 cells that have either an active or inactive CamK2α promoter. Previous studies have indeed demonstrated that not all CA1 neurons express CamK2α [33]. Interestingly, CamK2α expression has been shown to distinguish functionally distinct granule neurons in the olfactory bulb; however, such a distinction has not yet been documented in the hippocampus. The correlation between CamK2α expression and baseline cFos expression in CA1 neurons demonstrated in our study provides a basis for further investigations into the functional relevance of these two subpopulations in the hippocampus. The second example of diversity within a population revealed by our data is the presence of Tcf4+ and Tcf4− PC in the cerebellum. However, the ‘all’ or ‘nil’ Tcf4 expression pattern in PC could also be a result of two dynamic states of PC activity correlated with Tcf4 expression. In either case, the functional implication of Tcf4-positive and Tcf4-negative PC could reveal interesting cell biology of PC, warranting investigations in the future.

The other important revelation of our study is the elucidation of novel gene networks that potentially underlie mature neuronal structure and function. While Tcf4 has been studied in the embryonic brain and in heterozygous (Tcf4+/−) mouse models, these studies do not provide the required resolution to tease apart a potentially distinct role of Tcf4 in developing versus mature neurons. In this context, our transcriptomic dataset of adult-deleted Tcf4-KO FACS-sorted excitatory neurons is the first and only transcriptome dataset, as per our knowledge, that provides insights into the in vivo targets of Tcf4, specifically in adult brain neurons. A comparison of the transcriptome of mature neurons after acute loss of Tcf4 during adulthood (our RNA-Seq dataset) with the transcriptome of either embryonic Tcf4-KO P0 brain or adult CA1 cells from Tcf4-heterozygous animals (publicly available datasets from Li et al., Kennedy et al. and Phan et al.) indeed demonstrated that the targets of Tcf4 in embryonic brain are vastly distinct from its targets in mature neurons. While in embryonic brain Tcf4 targets cell-cycle-related genes affecting cell proliferation and differentiation, in the mature neurons of the adult brain Tcf4 regulates a distinct set of genes that predominantly participates in plasma membrane and microfilament-based processes.

The overall transcriptome of Tcf4-KO mature neurons alludes to a suppressive role of Tcf4 in the adult brain excitatory neurons. Several of the top targets of Tcf4 in mature neurons, such as aquaporin 1 and the angiotensin converting enzyme, are rarely studied in the context of neurons. In the same vein, suppression of ciliary processes related gene networks by Tcf4 in mature neurons, as revealed by ontology analysis, points to players and pathways that are poorly documented in mature neuronal function. Although further investigations will be required to validate and fully uncover the functional implications of the differentially regulated genes, examples of cilium regulation in neuronal function, such as in morphogenesis processes is already recognised [41, 42]. Furthermore, the physiological relevance of primary cilia in mature neuron was recently demonstrated in dentate GC through inducible deletion of ciliary component IFT20 in mature DG GC, which revealed enhanced LTP at mossy-fibre synapses and impaired contextual memory in animals with disrupted cilia in mature DG cells [43]. Our morphological observations demonstrate the potential for significant dendritic changes in adult brain neurons. Despite previous evidence for dendritic restructuring in the adult brain [44–46], dendritic plasticity in adult neurons remains a poorly studied subject. Given the continual expression of Tcf4 in adult neurons throughout the lifespan, it is possible that Tcf4 plays a role in maintaining the normal dendritic structure of adult neurons, while also providing a mechanism by which an adult neuron could alter structure, if need be, such as under pathological conditions and aging [47].

Overall, our study not only uncovers the functional role of the psychiatric disease-risk gene Tcf4 in mature neurons but also provides critical insights into the structure-function regulation of adult neurons, revealing involvement of previously unappreciated molecular pathways. The gene networks regulated by Tcf4 open up novel avenues of investigations for insights into the mechanisms that underlie mature neuronal regulation and homoeostatic functioning of the adult brain.

## Materials and methods

### Mice

All animals were housed, bred and used according to the protocols approved by the Institutional Animal Care and Ethics Committee. For conditional targeting of Tcf4 in CaMK2α-expressing excitatory neurons in the adult brain, Tcf4-*flox* line [31, 32] was crossed with CaMK2αCreERt2 line [29] to produce CaMK2αCreERt2; Tcf4^*fl/fl*^ (KO) or CaMK2αCreERt2; Tcf4^+/+^ (WT). For genetic tracking, Rosa-*flox*-STOP-*flox*-EGFP reporter line was further crossed on to CaMK2αCreERt2; Tcf4^*flox*^ and CaMK2αCreERt2; Tcf4^*wt*^ lines. For morphometric tracing, the Thy1-EGFP line M was crossed on to CaMK2αCreERt2; Tcf4^*flox or WT*^ lines. All animals were bred and kept at the institutional animal care and resource centre.

### Mice treatments

Tamoxifen (Sigma) was used at 5 mg/35 g body weight once a day for 7 days for CaMK2αCreERt2 induction in WT and KO adult animals at the age of P45–60. Same dose was used for genetic labelling in CaMK2αCreERt2; Tcf4^*fl/fl or WT*^; Rosa-*flox*-STOP-*flox*-EGFP and CaMK2αCreERt2; Tcf4^*fl/fl or WT*^;Thy1-EGFP line M.

### Immunostaining

Animals were anaesthetised with halothane followed by transcardial perfusion with PBS and 4% PFA. The brains were fixed in 4% PFA overnight followed by sectioning on Leica Vibratome to obtain 30 µm sections. Some animals were taken for dual experiments, where half brain was used for immunostaining. In these cases, half brains were directly fixed in 4% PFA for two overnights at 4 °C without transcardial perfusion. The floating sections were first blocked in 10% normal goat serum, 1% BSA, 0.1% triton and 100 mM glycine for 1 h at RT and stained with primary antibody (in 1% normal goat serum, 0.1% BSA, 0.1% triton and 100 mM glycine) overnight at 4 °C. Sections were then washed and incubated with fluorophore conjugated secondary antibody for 1 h at RT in dark, before mounting and imaging. Primary antibodies used were: CaMKII alpha (Invitrogen #13-7300), calbindin (SySy #214 005), parvalbumin (SySy #195 004), cFos (SySy #226 004), NeuN (Millipore # MAB377) and TCF4 (Abcam #ab217668).

### Confocal imaging and analysis

Sections were imaged using Leica SP8 confocal microscope. Images were analysed using Imaris and ImageJ. For Tcf4 protein expression characterisation, 63× objective images were taken. For cFos quantification, 20× and 40× images were used. Cell counts were normalised with the volume of CA1 for each image, ~10 hippocampal sections spanning rostral to caudal hippocampus were analysed per animal. Quantification plots were done both as per section and per animal, so as to demonstrate the actual spread of data within an animal’s brain. Non-parametric Wilcoxon test (per section counts) and parametric Student’s *t* test (per animal counts) were used for statistical analysis of cFos, since the cFos counts distribution in the hippocampus tested positive for normality test. All graphs were plotted using GraphPad Prism.

### Morphometry

CaMK2α-CreERt2; Tcf4 *wt* or *flox* mice were crossed with the sparsely expressing Thy1-GFP line M for enabling visualisation of the dendritic processes. For morphometric experiments, 120 µm sections were obtained following transcardial perfusion with PBS and 4% PFA. 63× objective imaging with optimised number of z slices was done for CA1 pyramidal neurons. The apical dendrites were manually traced using Neurolucida (2017, MBS Bioscience, Williston, VT, USA) followed by Sholl analysis using Neurolucida Explorer. Sholl radius interval was set at 5 μm. Three mice per genotype were analysed with 8–10 CA1 neurons per animal. Two-way ANOVA was used for statistical analysis. Spines were imaged using Leica SP8 confocal microscope at 100× magnification with 2× zoom. Spine characterisation was done manually using ImageJ on fragments of apical dendritic processes. Spine counts were normalised with the length of the dendritic fragment. Approximately 20–25 dendritic fragments per mouse were analysed for spine counts. Quantification plots were done both as per section and per animal, so as to demonstrate the actual spread of data within an animal’s brain. Student’s *t* test was used for statistical analysis based on positive normality test for CA1 spines.

### Behaviour experiments

For all behaviour experiments, the animals were housed in their regular individually ventilated cages in SPF facility with easy access to food and water with 14/10 h of light and dark cycles, respectively. Before the start of experiment, animals were individually handled for 5 min for 3 days. All analyses were done blind to genotype.

#### Social behaviour test

Social behaviour was conducted in a Y-maze apparatus as described previously [48]. Briefly, the Y-maze had two cylindrical holding areas attached to the two short arms for holding mouse or an inanimate dummy mouse. The long arm of the apparatus was used as the start arm, where the test animal was placed. The holding area at the end of the two short arms had smooth metal fence so that the test animal was easily able to view and interact with the animal in the holding space across the fence. On day 1, animals were acclimatised with the Y-maze arena in which experiment was to be performed. For this, the animals were released individually in arena for 5 min and then placed back into home cages. On day 2, the test was performed which was divided into two phases: a ‘dummy-test’ phase that was used to assess the innate sociability of the animals, and the ‘novel-mouse’ test phase that was used to assess social memory. In the ‘dummy’ phase, a dummy mouse was put in one of the holding cages of the two short arms, whereas a live mouse was put in the other arm. The test animal was released in the long arm and allowed to freely explore for 5 min. After the fixed exploration time of 5 min, the test animal was taken back to home cage for 2–3 min. After the ‘dummy phase’, the ‘novel-mouse’ test was done, in which the inanimate dummy mouse was replaced by a live unfamiliar mouse which was termed as ‘novel’ mouse, for the social behaviour experiment. The ‘novel’ and ‘familiar’ mice used in the training and test sessions were of the same gender as the test animal. The test animal was released in the long arm and allowed to explore in Y-maze for 5 min. Throughout the tests, the arena was cleaned with 70% ethanol before and after an animal’s entry to the arena. The ‘dummy’ and ‘novel-mouse’ test sessions were recorded with top head camera, and the videos were manually analysed for exploration time at each of the cages at the short arms. Exploration in ‘dummy phase’ was used as proxy for their sociability, and exploration time with the ‘novel mouse’ in comparison to the ‘familiar mouse’ in the ‘novel-mouse’ test was used as proxy for their social memory. For this, the time spent in active sniffing and climbing towards the caged animal or dummy was counted as exploration. Head not facing the caged animal/dummy was not considered as exploration. Statistical analysis was done using Mann–Whitney test.

#### Contextual-fear-memory test (CFC)

Previously published protocol was followed for CFC [49]. The CFC apparatus which was used for the training and context test was a rectangular chamber with an electrifiable grid floor, an odour source: a tray with 70% ethanol under the grid floor, a light source and a calibrated shock generator. On day 1, animals were acclimatised in the CFC chamber in which experiment was to be performed. For this, the animals were released individually in the chamber for 5 min and allowed to freely explore. After 5 min, the animal was placed back into their home cage. On day 2, the training day, the animal was introduced in the CFC chamber for 3 min. At the end of 3 min a 2 s foot shock of 0.9 mA was given, after which the animals was allowed another 15 s in the chamber, before being taken back to their home cage. On day 3, the test day, the mice were reintroduced in same shock context and allowed to explore undisturbed for 3 min. No shock was given on this day. Movement and/or freezing on both training and test sessions were recorded by a camera fitted inside the chamber. The amount of time when the animal did not move at all was called the ‘freezing time’. Analysis of freezing behaviour was performed using ANY-maze software. Percent time spent freezing out of the total time in chamber was calculated to use as a proxy for the animal’s contextual-fear memory. Statistical analysis was done using Mann–Whitney test.

#### Open-field test

OFT was performed as described previously [49]. The ‘open-field arena’ was a square shaped box (38 × 38 cm) with a top head camera covering the entire arena’s field of view. The animal was introduced into the arena and allowed to explore for 10 min. The distance travelled and speed of the animals were used to assess locomotor function. The analysis of distance travelled and speed was performed using ANY-maze software. Statistical analysis was done using Mann–Whitney test.

### Electrophysiology

CA1 neurons were identified in hippocampal slices under an upright differential interference contrast microscope (BX1WI microscope, Olympus) using a 40× objective (water immersion lens, 0.9 numerical aperture, LUMPLFLN, 40×). 2–4 MΩ pipettes were pulled from thick-walled borosilicate glass capillaries on a P-1000 Flaming Micropipette Puller (Sutter Instrument). The pipettes were filled with an internal solution of the following composition for whole-cell current-clamp recordings (in mM): 120 potassium gluconate, 20 KCl, 0.2 EGTA, 4 NaCl, 10 HEPES buffer, 10 phosphocreatine, 4 Mg-ATP and 0.3 Na-GTP (pH 7.4 and 295 mOsm). For sEPSC recordings, caesium based internal of following composition was used (in mM): 130 CsOH, 13 gluconate, 5 NaCl, 10 HEPES, 1 EGTA, 2 MgCl2, 2 Mg-ATP, 0.5 Na-GTP and 10 phosphocreatine. The solution was filtered using 0.22 μM filter before it can be filled in the glass pipette for patching. After entering into whole-cell mode, cells were held for at least 3–5 min before recording was started. This is needed for stabilisation of the cells. CA1 cells were recorded if they had a resting potential of ≤55 mV. Series resistance and IR were continuously monitored during the voltage clamp protocols, and the cell was discarded if these parameters changed by >20%. From a slice maximum of four cells were used for recording. All analysis was done in MATLAB or Clampfit. Custom written codes were used in MATLAB for analysis of *F*/*I* curve, *I*_*h*_ currents, RMP and rheobase. IR was found plotting IV curve for a cell using Clampfit. All analyses were done blind to genotype.

### Neuron sorting by FACS

For single-cell suspension preparation was same brain was scooped, followed by rapid dissection of desired regions in chilled Hibernate-A (BrainBits #HACA) media. For FACS, hippocampus was micro-dissected, and tissue was minced in digestion media (30 μl/ml papain [Sigma Aldrich #P4762], 10 μg/ml DNase I [Roche #4716728001] prepared in HA) followed by 30 min incubation with mild shaking at 37 °C. Tissue was triturated using fire-polished glass pipettes and centrifuged at 1200 rpm for 10 min. Myelin was removed using 32% isotonic Percoll^TM^ (GE Healthcare #GE17-0891-01). Pellet obtained was stained with CD45-PerCP5.5 and CD11b-APC-Cy7 to differentiate myeloid and neural cells. DAPI was used to identify dead cells. CD11b− CD45− cells were sorted for GFP+ cells, which were directly collected in RLT Plus (Qiagen #1053393) followed by RNA isolation using RNeasy Micro Kit (Qiagen #74004). Cells were isolated from the hippocampus of 3 WT and 3 KO brains; however, the process of FACS-sorting adult neurons yielded low cell counts for one pair of WT/KO brains, therefore we had to pool this pair with another pair of WT/KO cells, and the third pair of samples from individual WT and KO brain was used as the second sample, resulting in two samples per genotype for RNA sequencing.

### RNA sequencing

GFP+ cells were sorted from 3 WT and 3 KO brain HPC, but 2WT and 2KO were pooled as one sample in each genotype because of low cell yield. Sample QC, library preparation and RNA sequencing were done by Next Generation Sequencing facility at NCBS. RNA quality was assessed using Bioanalyzer. cDNA libraries were prepared by using NEB stranded mRNA library prep kit as per manufacturer’s instructions. PolyA selection method was used to enrich mRNA. Library quality was analysed with Bioanalyzer. Next-generation sequencing of libraries was performed on Illumina HiSeq 2500 platform for 1 × 100 bp at ~20–25 million reads per sample.

### RNA sequencing data analysis

The public server at usegalaxy.org was used to analyse the sequencing data. Briefly, after cutting the adaptor sequence and initial quality check for sequence reads, HISAT2 was used to align the reads to mus musculus mm10 reference genome, and feature counts were generated. For differential gene expression, the DESeq2 tool was used and the output was annotated using ‘annotateMyID’ feature in Galaxy. The differential gene expression output was used for volcano plot using R. Normalised feature counts were used to plot heatmap using the webtool ‘heatmapper’ [50]. Enriched ontology cluster analysis was done using the public webtool at metascape.org [51]. The enriched ontology clustering was performed through the Metascape feature as described in detail on their website. Briefly, all statistically enriched GO terms were used for hierarchically clustering based on Kappa statistical similarities among their gene memberships, and 0.3 kappa score was applied as the threshold to cast the tree into term clusters. A subset of representative terms from this cluster was used to convert them into a network layout. Terms with a similarity score > 0.3 were linked by an edge (the thickness of the edge represents the similarity score). The network was visualised with Cytoscape (v3.1.2) with ‘force-directed’ layout and with edge bundled for clarity. One term from each cluster was selected to have its term description shown as label. For Venn diagram, the public webtool ‘venny 2.1’ was used. Fisher’s exact test was used for testing significance of overlapping genes.

## Supplementary information

Supplemental Table 1

Supplemental Figure
