# Supplementary material for: Adult brain neurons require continual expression of the schizophrenia-risk gene Tcf4 for structural and functional integrity
Source: Transl Psychiatry. 2021 Sep 25;11:494. doi: 10.1038/s41398-021-01618-x (PMC8464606; doi:10.1038/s41398-021-01618-x)
Supplement: Supplementary file 2 — Supplemental Figure [file 41398_2021_1618_MOESM2_ESM.docx]

**Supplemental Information**

**Supplemental Figures:**

**Figure S1**

**
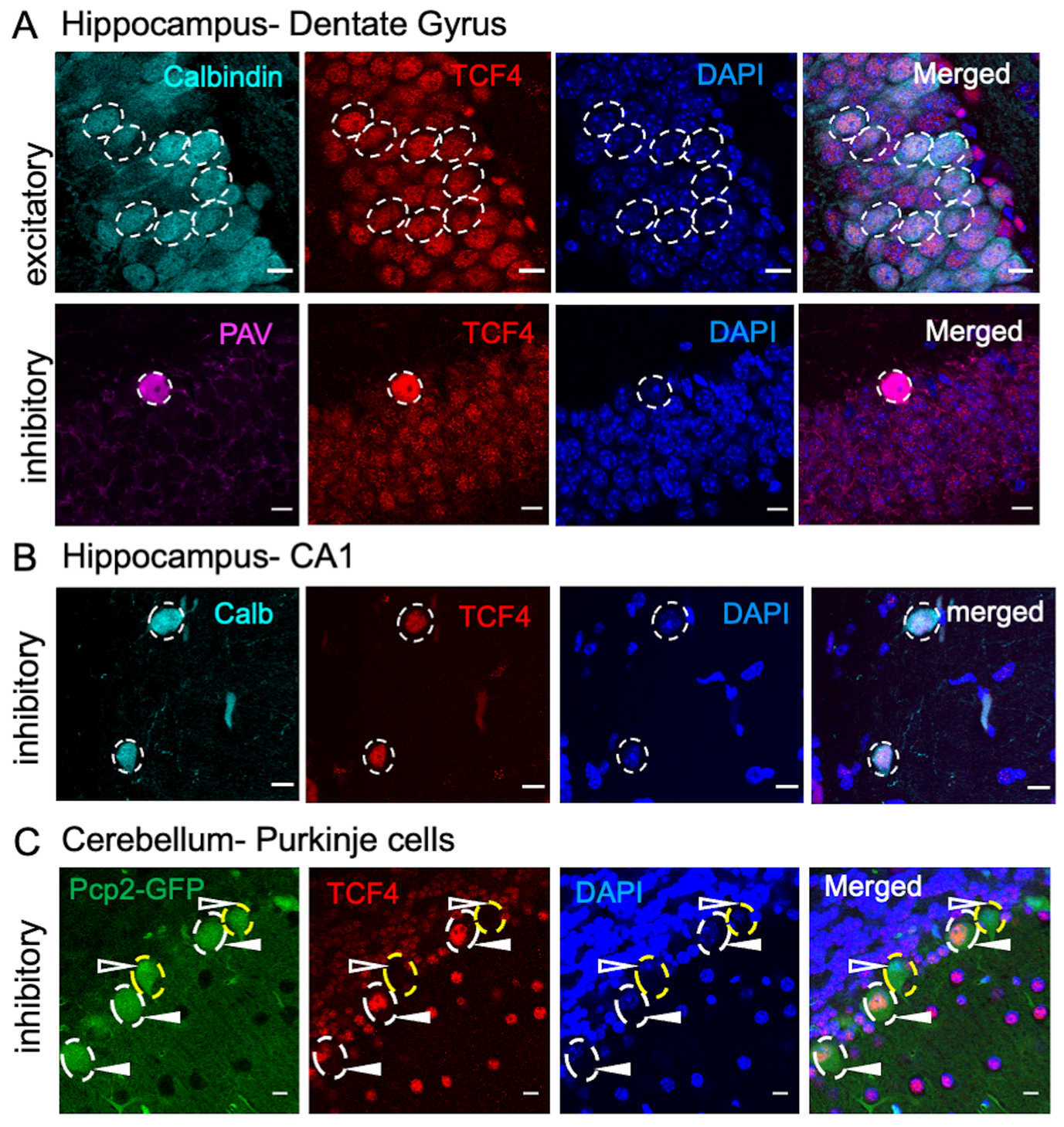
**

**Fig S1: Tcf4 protein expression in mature neurons of the adult brain**

A. Representative immunofluorescence image showing high Tcf4 protein expression in the nucleus of excitatory (Calbindin+) and inhibitory (Parvalbumin+) neurons in dentate gyrus of the hippocampus. B-C. Representative immunofluorescent image showing Tcf4 protein expression in the nucleus of Calbindin+ inhibitory neurons in CA1 region of the hippocampus (B) and PCP2+ Purkinje cells in the cerebellum (C). Representative of 3-5 adult mice, Scale bar= 10µm.

**Figure S2**

**
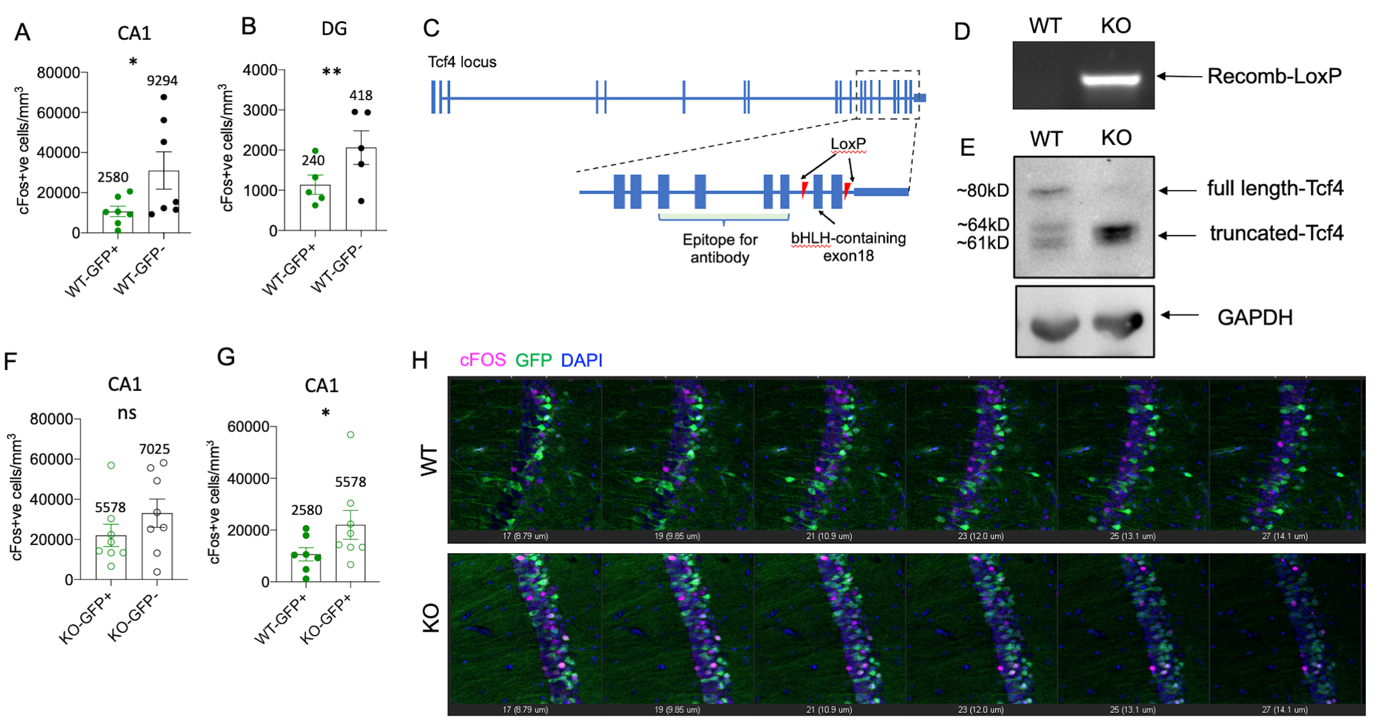
**

**Fig S2: Tcf4-deletion in adult CA1 neurons increases c-Fos activity**

A-B. Per animal cFos cell counts from Fig 2A-B, +/- SEM value shown on the graph, C. Schematic of the Tcf4-locus depicting the LoxP sites flanking the bHLH domain at the C-terminus, and the N-terminal region where epitopes recognized by the Antibody reside, D. Recombination PCR confirming Tcf4-deletion; PCR band detecting Tcf4-LoxP recombined locus only in KO, E. Western blot showing Tcf4-deletion in adult brain in the inducible-knock-out (iKO) mice. F-G. Per animal cFos cell counts from Fig 2D-E, (each dot represents an animal, CA1: 7WT, 8KO and DG: 5 WT, 5KO animals), paired t-test, * p<0.04, **p=0.002. +/- SEM is depicted as numbers on the graphs. H. Z-stack single plane images of the middle optical slices for cFos-staining from Fig 2C demonstrating uniform cFos antibody penetration and staining in the middle layers of the brain sections. The optical slice number and depth of the section in micron can be seen in each frame’s label.

**Figure S3**

**
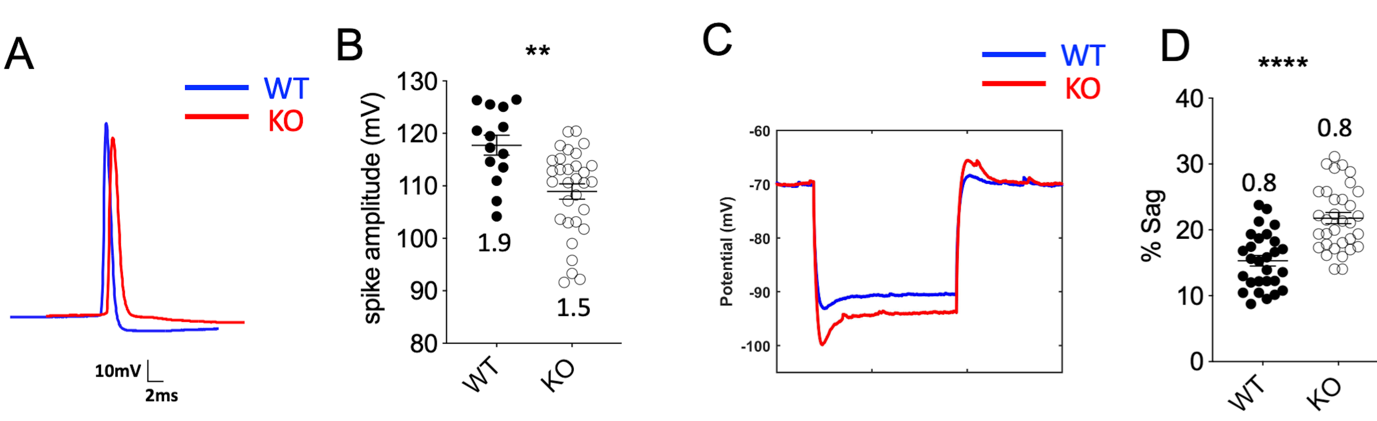
**

**Fig S3: Tcf4 deletion in adult excitatory neurons results in hyperexcitability**

A. Representative spike amplitude trace, B.Spike amplitude quantification;+/- SEM value shown on the graph, C.Representative trace for IH current sag after hyperpolarizing pulse, D. quantified % Sag +/- SEM value shown on the graph, Recordings from 27 neurons from WT and 33 neurons from KO brains, representing ~5-6 mice per genotype. Mann Whitney test: **p<0.001, ****p<0.0001).

**Figure S4**

**
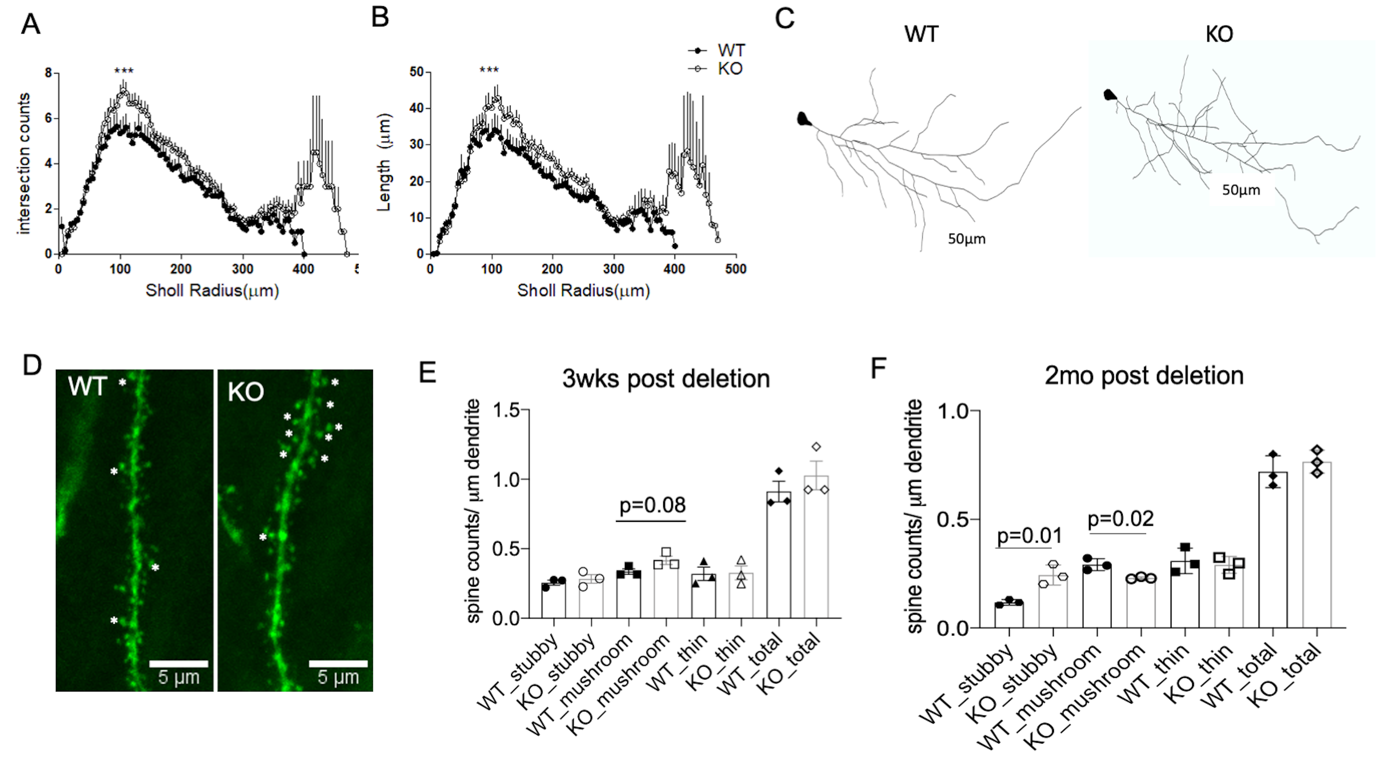
**

**Fig S4: Tcf4 deletion in adult excitatory neurons results in increased dendritic branching**

A-B. Sholl analysis of apical dendrite of CA1 neurons, showing number of intersection (A) and length (B) per sholl radii 3 weeks post deletion, 23 WT and 33 KO neurons from 3WT and 4KO mice, Two-way ANOVA, ***p< 0.0001, error bar represents SEM, C. Representative traces of apical dendrites of CA1 neurons in ‘A-B’, D. Representative image showing spines in CA1 neurons 3 weeks post deletion, white asterisks shows mushroom spines, E-F. Spine analysis of CA1 apical dendrites at 3wks (+/-SEM: 0.01-0.05) (E) and 2mo post deletion (+/-SEM: 0.003-0.02) (F), each dot represents counts from an animal. Unpaired t-test was used for statistical analysis.

**Figure S5**

**
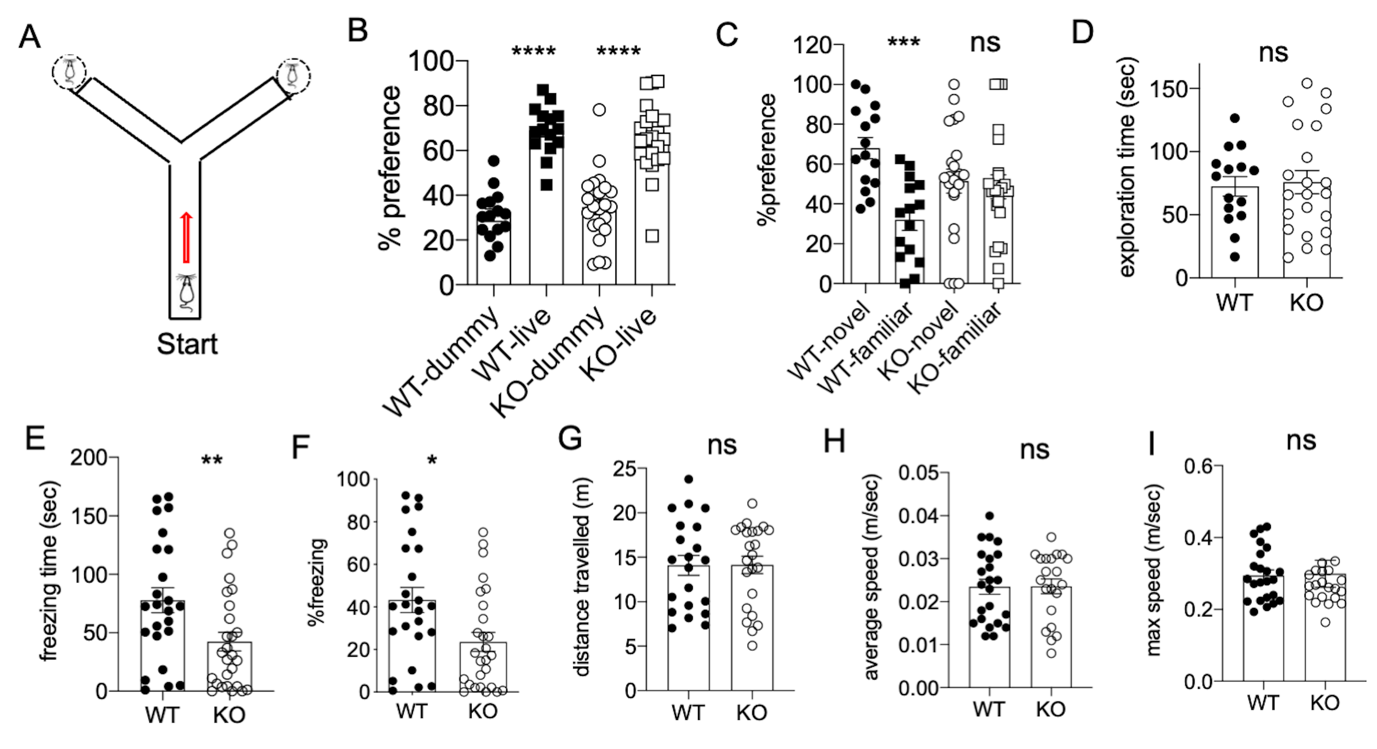
**

**Fig S5: Tcf4 deletion in adult excitatory neurons results in cognitive deficits**

A. Diagram of Y maze used for social behavior test, B. Performance in Sociability (+/-SEM WT: 2.8, KO: 3.3), C. Social memory test (+/-SEM WT: 5.2, KO: 6.0), showing normal sociability but deficient social memory for Tcf4-KO animals. D. Total exploration time during the social memory task showing no deficit in exploration (+/-SEM WT: 7.7, KO: 9.2); n= 15 WT, 22 KO animals. E-F. Performance in contextual fear memory task showing deficient memory in KO animals, as indicated by decreased freezing time, +/-SEM; WT:10.7, KO: 8.0, (E) and % freezing time of total time spent in chamber; +/-SEM, WT: 5.8, KO:4.4, (F) (n=24 WT, 27 KO animals), G-I. Open-field test showing no difference between WT and KO animals in locomotion, +/-SEM: total distance- WT:1.1, KO: 0.9, avg speed- WT & KO: 0.001, max speed- WT:0.01, KO:0.03, (n=21 WT, 23 KO animals), Each dot represents an animal (Mann-Whitney test, ****p<0.0001, ***p=0.0001,**p=0.001, *p=0.019, ns= non-significant).

**Figure S6**

**
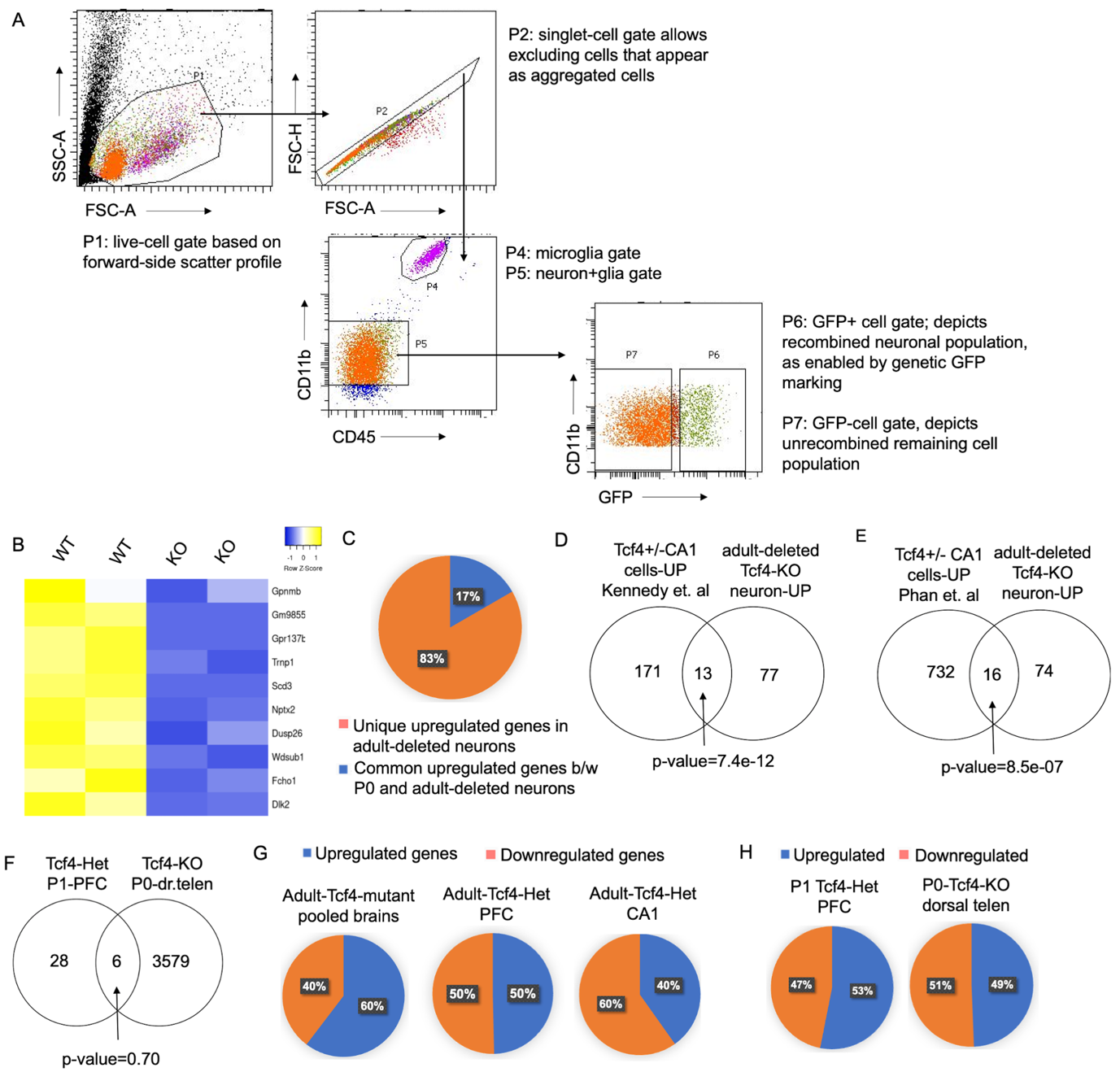
**

**Fig S6: Tcf4 targets are distinct in adult excitatory neurons when compared to embryonic brain**

A. Gating strategy used for FACS-sorting GFP+ve mature neurons from adult brain hippocampus. The GFP+ve fraction, depicting genetically-labelled WT or KO excitatory neurons were sorted for RNA-Seq analysis, 3WT and 3KO brain HPC (2WT and 2KO were pooled as one sample in each genotype because of low cell yield), B. Heatmap showing downregulated genes for the FACS-sorted GFP+ve hippocampal neurons, C. Pie chart showing percent overlap in up and down-regulated genes between adult-deletedTcf4-KO neurons and P0 Tcf4-KO telencephalon, D-E, Venn diagram showing minimal overlap of upregulated genes between adult-deleted Tcf4-KO neurons and heterozygous KO (Tcf4+/-) adult CA1 cells from two different studies (Kennedy et. al and Phan et. al).Fisher’s exact test used for statistics,F. Comparison of P1-Tcf4-het PFC and P0 Tcf4-KO telencephalon shows insignificant overlap, G-H. Percent Up and Down-regulated genes in adult (G) and neonatal (H) brains shows bi-direction regulation of Tcf4 on overall transcription.
